# Allergenic Potential of Common Hops (*Humulus lupulus* L.) in the Context of Cross-Reactions with Pollen Allergens

**DOI:** 10.3390/nu16213626

**Published:** 2024-10-25

**Authors:** Dorota Piasecka-Kwiatkowska, Kinga Blacharska, Ewa Springer

**Affiliations:** 1 Department of Food Biochemistry and Analysis, Poznan University of Life Sciences, 60-623 Poznan, Poland; 2 Specialized Non-Public Health Care Facility Alergologia Plus, Allergy Diagnostics and Therapy Center, 60-693 Poznan, Poland

**Keywords:** common hops (*Humulus lupulus* L.), supplements, immunoreactivity, cross-reactivity, pollen allergens

## Abstract

Background: Common hops (*Humulus lupulus* L.) play a key role in brewing, providing the bitterness, flavor, and aroma of beer, and are widely used in supplements for their antibacterial, anti-inflammatory, and antioxidant properties. However, despite their broad applications, the allergenic potential of common hops remains underexplored, particularly when compared to the closely related *Humulus japonicus*. This preliminary study aimed to investigate the allergenic potential of common hops and their potential cross-reactivity with common pollen allergens. Methods: The immunoreactivity of hop stalks, leaves, and cones was assessed using antibodies against major allergens from birch (Bet v1a), mugwort (Art v1), and timothy grass (Phl p5b), as well as three sera from pollen-allergic patients. Slot Blot analysis was performed using phosphate-buffered saline extracts from the stalks, leaves, and cones of three hop cultivars, while Western Blotting followed SDS-PAGE protein separation. Results: The results revealed significant immunoreactivity in native hop proteins, with diminished reactivity observed in denatured proteins. Cross-reactivity between hop proteins and major pollen allergens was confirmed, indicating that hop proteins may contribute to allergic sensitization in pollen-sensitive individuals. Conclusions: These findings underscore the potential allergenic risks associated with the consumption or exposure to hop-containing products.

## 1. Introduction

Hops (*Humulus lupulus* L.), a member of the Cannabaceae family and the Rosales order, are represented by three species: the common hop (*Humulus lupulus* L.), the Japanese hop (*Humulus japonicus*), and *Humulus yunnanensis*. Of these, the common hop is especially significant as a key ingredient in the brewing industry, where its cones contribute bitterness, flavor, and serve as a natural preservative in beer [1]. In addition to their role in brewing, hops are known for their sedative properties, often used in dietary supplements to promote sleep and reduce anxiety [2,3,4]. Beyond these uses, common hops are recognized for their various health benefits, including antimicrobial, anti-inflammatory, estrogenic, cardioprotective, and anticancer properties [5,6,7,8,9]. However, they are also recognized as potential allergens, particularly for individuals working in breweries, although research on this issue remains limited [10,11,12].

In contrast, the closely related Japanese hop (*Humulus japonicus*) is a well-established allergen, commonly associated with autumn pollinosis in East Asia, especially in China, Japan, and Korea [13,14,15,16]. More than ten immunoglobulin E (IgE)-reactive protein components, ranging from 11 to 70 kDa, have been identified in Japanese hop pollen. Of these, two major allergens—Hum j 1 (10 kDa) and Hum j 6 (10–15 kDa)—are officially recognized by the WHO/IUIS Subcommittee on Allergen Nomenclature [17]. Despite the close taxonomic relationship between *Humulus lupulus* L. and *Humulus japonicus*, no significant cross-reactivity between these species has been reported [18].

Allergic diseases, particularly those induced by pollen, have become an increasing health concern in modern society [19]. Pollen allergies often result from exposure to more than one allergen source, which complicates diagnosis and treatment [20]. According to the European Academy of Allergology and Clinical Immunology (EAACI), 60% of cases of food allergy in both adults and children are linked to coexisting inhalant allergies [21]. The complexity of allergic responses often arises from cross-reactions between seemingly unrelated allergens, a phenomenon driven by panallergens. Panallergens are structurally conserved proteins that share similar epitopes across different plant species, which can cause cross-linking of IgE on mast cells, triggering an allergic response. Significant panallergen families include profilins, polcalcins, non-specific lipid transfer proteins (nsLTP), and pathogenesis-related proteins class 10 (PR-10). Profilins and polcalcins are primarily found in tree, grass, and weed pollens, while nsLTP are present in both tree and weed pollens, and PR-10 proteins are predominantly located in tree pollen. All of these panallergens contribute significantly to cross-reactivities between different pollens and between pollen and food allergens [22]. According to some studies [23], up to 50% of individuals with pollen allergies are sensitized to at least one of two major panallergens: profilin or polcalcin.

Profilins, small proteins (12–15 kDa), are ubiquitous in all eukaryotic cells and have highly conserved amino acid sequences, with at least 75% identity between species, making cross-reactivity between profilins common. Individuals sensitized to one profilin can react to similar proteins from other sources. Notable profilin allergens include birch Bet v 2 (14–15 kDa), mugwort Art v 4 (14 kDa), and timothy grass Phl p 12 (14 kDa) [24,25,26].

Polcalcins, another significant family of panallergens, are calcium-binding proteins involved in signal transduction. With high sequence identity (around 77%) and molecular masses of about 8 kDa, polcalcines are widespread in tree, grass, and weed pollen. Their characteristic IgE epitopes, modulated by calcium-binding, become accessible only in their calcium-bound open conformation. Representative polcalcin allergens include birch Bet v 4 (7–8 kDa), mugwort Art v 5 (10 kDa), and timothy grass Phl p 7 (6–8.5 kDa) [25,26].

Non-specific lipid transfer proteins (nsLTPs) which play a key role in plant defense, are present in all plant organs and are significant in pollen–food cross-reactivity. The International Union of Immunological Societies (IUIS) Allergen Nomenclature Subcommittee has identified 46 nsLTP allergens from various fruits, vegetables, nuts, seeds, and pollen. These allergens are classified into two groups: nsLTP1 (9–10 kDa) and nsLTP2 (6–7 kDa), with most allergenic nsLTPs falling under type 1. Representative nsLTP allergens include peach Pru p 3 (10 kDa), apple Mal d 3 (9 kDa), and cherry Pru av 3 (10 kDa), along with pollen allergens such as mugwort Art v 3 and ragweed Amb a 6 [17,22,25].

The PR-10 protein family is another major group of panallergens, with molecular weights ranging from 15 to 18 kDa. These proteins are widespread in vascular plants and are characterized by conserved structures containing a large ligand-binding cavity. They are involved in plant defense and are implicated in allergic reactions to pollen. Key PR-10 allergens include the major birch pollen protein Bet v 1 (17 kDa), alder Aln g 1 (18 kDa), and hazel Cor a 1 (17 kDa) [25,27,28,29].

The presence of panallergens can lead to border IgE responses, increasing the risk of multiple allergies and severe symptoms [22,28,30,31]. Moreover, pollen allergies can contribute to the development of food allergies, with prevalence rising as sensitization to inhalant allergens increases [28,32,33,34]. In some cases, these allergies may also contribute to oesophageal and gastrointestinal diseases in the context of allergic gastrointestinal processes [35]. Additionally, inhalant allergens like pollen can trigger respiratory conditions, such as asthma, which affects millions worldwide. In 2022, the World Health Organization (WHO) reported over 260 million people suffering from asthma, with more than 450,000 associated deaths annually [36].

With the increasing prevalence of pollen allergies and the growing consumption of bioactive plant-based foods and supplements, identifying potential cross-reactive allergens in commonly used plants is essential. Prolonged and frequent exposure to certain allergens increases the risk of cross-reactivity, as higher levels of specific IgE antibodies are associated with a greater likelihood of allergic reactions. IgE stimulated by cross-reactive inhalant allergens can lead to diverse allergic responses, including food allergies, which may manifest as mild local symptoms or even severe systemic reactions after the first exposure to a cross-reactive food allergen [21].

Given the rising prevalence of pollen allergies and the growing incorporation of bioactive plant components into diets and supplements, it is crucial to assess the potential for cross-reactions between unconventional plant ingredients and well-established pollen allergens. This preliminary investigation aims to evaluate the immunoreactivity of common hops (*Humulus lupulus* L.) with immunoglobulin G antibodies, recognizing major allergens for birch, mugwort, and timothy grass, as well as with immunoglobulin E from individuals sensitized to these pollen allergens. The findings will provide critical insights into the safety of common hops, particularly for consumers with pollen allergies, and contribute to better understanding the potential risk associated with unexpected cross-reactive allergens in dietary and environmental exposure.

## 2. Materials and Methods

### 2.1. Materials

The immunoreactive properties of common hop (*Humulus lupulus* L.) stalks, leaves, and cones of three Polish cultivars Marynka, Lubelski, and Magnum from the 2022 harvest were tested with commercial polyclonal antibodies, all prepared with recombinant protein (CUSABIO Technology LLC, Huston, TX, USA), recognizing the main allergens of birch Bet v 1a (CSB-PA322213HA01BSS), mugwort Art v 1 (CSB-PA774628HA01AOH) and timothy grass Phl p 5b (CSB-PA671435AA01EUQ). The CUSABIO antibodies were diluted in TBS buffer (20 mM Tris, 500 mM NaCl, pH = 7.4) with 1% of BSA at a 1:2000.

Immunoreactivity was also assessed with sera from allergic patients diagnosed with pollinosis, primarily to birch, timothy, and grass pollens. The study was conducted on archived and fully anonymous serum samples obtained from patients of the Specialized Non-Public Health Care Facility (SNZOZ) Alergologia Plus, Allergy Diagnosis and Therapy Center in Poznań (Poland). The collection and processing of blood for laboratory tests are part of the standard diagnostic and therapeutic activities of a unit registered in the Polish health care system. In addition, the patients provided explicit consent for the use of their serum in the scientific research. Since these activities do not constitute a medical experiment, approval from the Bioethics Committee was not required. The allergenic classes of the sera were specified based on Polycheck^®^ tests (Biocheck GmbH, Vorbergweg Germany), which measure sIgE levels, with a cut-off value of 0.35 kU/L considered a positive result (Class 0: <0.35 kU/L; Class 1: 0.35–0.7 kU/L; Class 2: 0.7–3.5 kU/L; Class 3: 3.5–17.5 kU/L; Class 4: 17.5–50 kU/L; Class 5: 50–100 kU/L; Class 6: >100). The different specific Polycheck^®^ kits used varied depending on the patients’ medical interviews and diagnoses. The panels included inhalation and food panels. The measurements were conducted at an accredited laboratory within the Diagnostyka Group in Poznań. The allergenic characteristics of the patients’ sera used in research are presented in Table 1. Before use, the sera were diluted in TBS-1%BSA buffer at a 1:20 dilution.

Immunocomplexes were detected using secondary antibodies conjugated with alkaline phosphatases. For CUSABIO antibodies, a mouse monoclonal anti-rabbit IgG γ-chain specific (A2556 Sigma-Aldrich, St. Luis, MO, USA) was used, while for sera, a monoclonal anti-human IgE (A18802 Invitrogen, Waltham, MA, USA). Both secondary antibodies were diluted in TBS buffer (pH 7.4) with 1% BSA and 0.05% Tween 20, at 1:175,000 and 1:2000 dilutions, respectively.

### 2.2. Protein Slot Blotting

Slot Blotting was carried out using a Slot Blotter (Geneflow, Lichfield, England, UK). The procedure followed was the same as previously described by Siekierzynska et al. [37] with the exception of the antibodies used (described above Section 2.1). Proteins from the crushed lyophilized hop samples (cones, leaves, stalks) of the three cultivars (Marynka, Lubelski, Magnum) were extracted using 10 mM PBS buffer at a ratio of 1:20. The samples were mixed and shaken at room temperature (20 °C) for 1 h, then centrifuged (15,000× *g*, 30 min) and stored at −20 °C. Extracts from each sample were prepared in triplicate. Protein concentration in the extracts was determined according to the Bradford method [38]. The analysis was conducted at a wavelength λ = 595 nm using a UV-Vis spectrophotometer (SP 8001 Metertech Inc., Taipei, Taiwan). 

The significance of the differences in protein concentrations extracted from the stalks, leaves, and cones of the three cultivars was determined using one-way analysis of variance (ANOVA) followed by Tukey’s post hoc test, with a significance level of *p* = 0.05. The protein concentrations of the extracts are presented in Table 2.

### 2.3. SDS-PAGE Electrophoresis

Proteins from the cones, leaves, and stalks of the three cultivars were separated using polyacrylamide gel electrophoresis under denaturing conditions [39] with the Bio-Rad Mini-PROTEAN system. A 0.5 g of grounded lyophilized sample was mixed with 78 µL of distilled water and 20 µL of lysis buffer containing 7 M urea, 2 M thiourea, 4% *w*/*v* CHAPS, 40 mM DTT, and protease inhibitor mix (GE Healthcare Bio-Sciences, Uppsala, Sweden). Protein concentration was determined using a 2-D Quant Kit (GE Healthcare Bio-Sciences, Piscataway, NJ, USA). A sample volume equivalent to 6 µg of protein and molecular marker ranging from 7 to 250 kDa (EURx E3215-01, EURX Ltd., Gdańsk, Poland) was loaded onto the gel. The separation was performed at 8 °C with a constant voltage of 70 V in 4% thickening gel for 30 min followed by 170 V in 12% resolving gel for 60 min. The gels were stained with Coomassie brilliant blue, scanned, and analyzed with CLIQS software ver. 1.1 (TotalLab Quant, Newcastle-Upon-Tyne, UK).

### 2.4. Western Blotting

In the first step, the samples were separated using 12% polyacrylamide gel electrophoresis under denaturing conditions with the BIO-RAD Mini-PROTEAN system (Los Angeles, CA, USA). Then, the proteins were transferred to a polyvinylidene difluoride (PVDF) membrane (Immobilon-P, 0.45 µm, Merc Millipore Ltd., Burlington, MA, USA) using the semidry Amersham Biosciences TE 77 PWR system (UK), with a constant current of 0.9 mA/cm^2^. Next, the membranes were blocked with the TBS-BSA buffer (pH = 7.4) for 45 min. Then, diluted sera or CUSABIO antibodies were applied for a 90 min incubation at room temperature. After five washes, the membranes were incubated for 90 min with an alkaline phosphatase-conjugated antibody. The membranes were washed again five times, and then the substrate BCIP/NBT (5-bromo-4-chloro-3′-indolyphosphate and nitro blue tetrazolium, Calbiochem, San Diego, CA, USA) was applied for 20 min. The reactions were stopped with water, the membranes were dried, and analysed with the CLIQS program ver. 1.1. (TotalLab Quant, Newcastle-Upon-Tyne, UK).

## 3. Results

### 3.1. SDS-PAGE Electrophoresis

**Figure 1:**
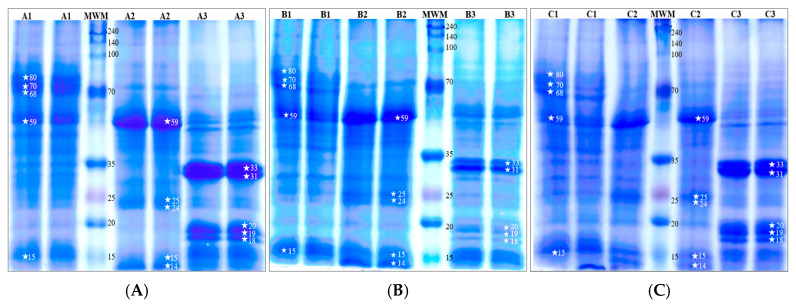
SDS-PAGE separation profiles with Coomassie blue staining of cones, leaves, and stalks from three common hop cultivars: (**A**) Marynka, (**B**) Lubelski (**C**) Magnum. Plant parts are labelled as follows: 1—stalk, 2—leaves, 3—cone. MWM represents the molecular weight marker. Asterisks (*) denote characteristic protein fractions, with the corresponding molecular weights (in kDa) indicated by the numbers.

The protein profiles of cones, leaves, and stalks from the three hop cultivars (Marynka, Lubelski, Magnum) were analysed using SDS-PAGE electrophoresis, and the resulting patterns are shown in Figure 1. Significant differences in protein profiles were observed between the plant parts across all cultivars. The stalks contained proteins with the highest molecular weights, with three predominant fractions around 80 kDa, 70 kDa, and 68 kDa, along with notable proteins at approximately 59 kDa and 15 kDa.

In contrast, the leaves were dominated by a protein fraction of about 59 kDa, with additional distinct bands at 25 kDa, 24 kDa, 15 kDa, and 14 kDa. The cones showed fewer protein bands at approximately 33 kDa, 31 kDa, 20 kDa, 19 kDa, and 18 kDa. These results highlight the distinct protein composition of each plant part, with stalks containing larger molecular weight proteins and cones exhibiting smaller molecular weight fractions.

### 3.2. Assessment of Hop Immunoreactivity Using Slot Blot

**Figure 2:**
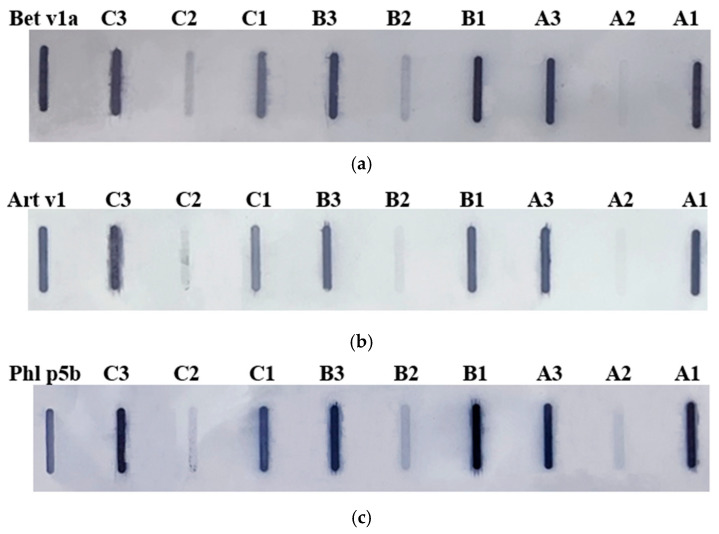
Membranes from the Slot Blot analysis of hop protein samples with commercial antibodies: (**a**) anti-Bet v 1a (birch), (**b**) anti-Art v 1 (mugwort), (**c**) anti-Phl p 5b (timothy grass). Cultivars: A—Marynka, B—Lubelski, C—Magnum. Plant part: 1—stalk, 2—leaves, 3—cone. Recombinant protein standard: Bet v 1a, Art v 1, Phl p 5b.

The immunoreactivity of hop proteins was evaluated using the Slot Blot technique, employing antibodies specific to major pollen allergens from birch (Bet v 1a), mugwort (Art v 1), and timothy grass (Phl p 5b). As shown in Figure 2, all hop protein extracts reacted to the allergen-specific antibodies, but differences in immunoreactivity were observed between the plant parts. Proteins from stalks and cones consistently exhibited higher immunoreactivity compared to those from leaves. Interestingly, no significant differences in immunogenicity were detected between the three hop cultivars.

**Figure 3:**
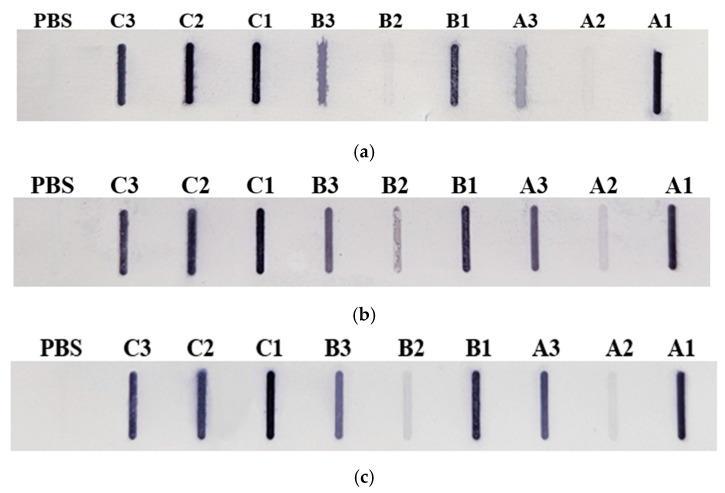
Membranes from the Slot Blot analysis of hop protein samples with patient sera: (**a**) serum 1, (**b**) serum 2, (**c**) serum 3. Cultivars: A—Marynka, B—Lubelski, C—Magnum. Plant parts: 1—stalk, 2—leaves, 3—cone. PBS—saline buffer.

In addition, hop protein extracts were incubated with sera from patients diagnosed with pollen and food allergies (Figure 3).

Similar to the results obtained with commercial antibodies, the leaves of the Marynka and Lubelski cultivars showed low IgE immunoreactivity compared to the stalks and cones, while the Magnum cultivar leaves exhibited strong immunoreactivity, comparable to the reactivity observed in cone and stalk extracts. Densitometric analysis confirmed that the cone extracts showed the highest overall immunoreactivity, regardless of the serum used.

### 3.3. Identification of Immunoreactive Fractions by Western Blotting

**Figure 4:**
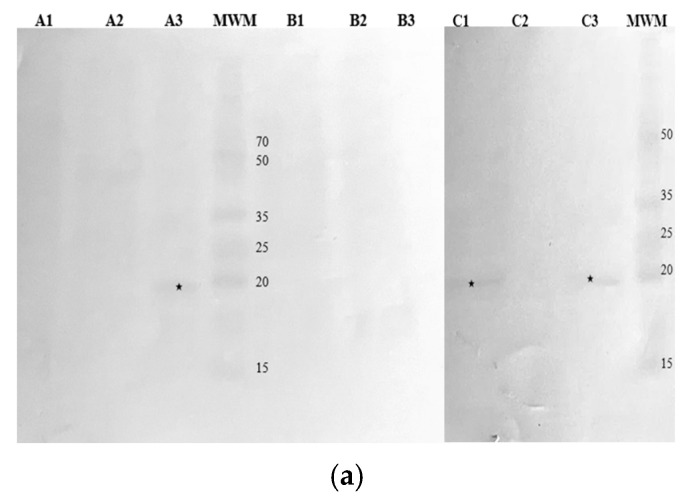

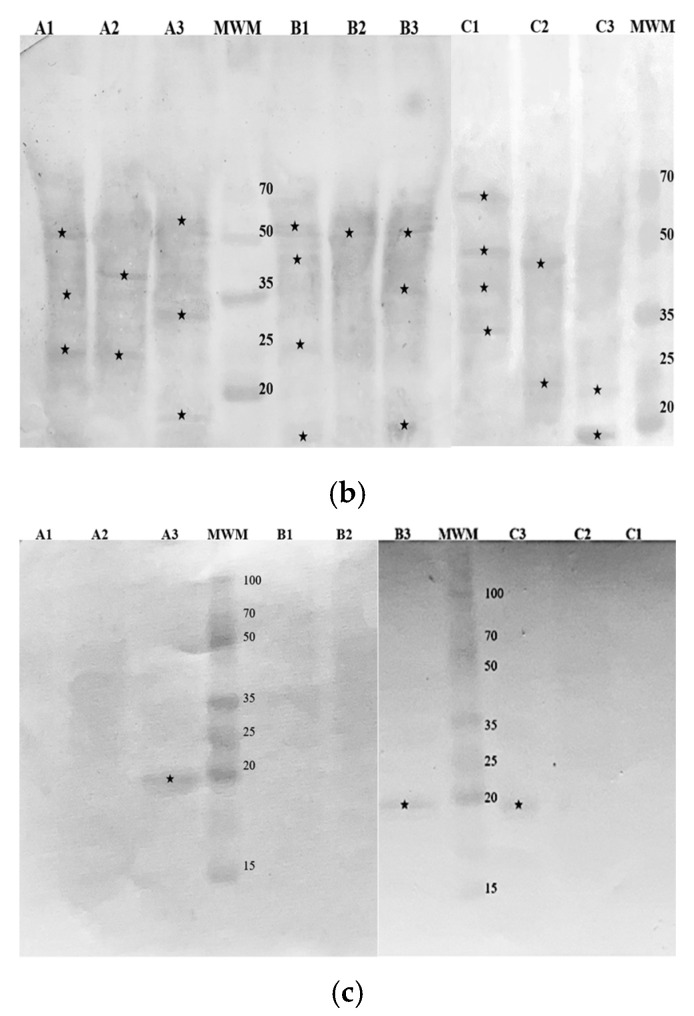
Membranes obtained after immunoblotting of hop samples with commercial antibodies recognizing: (**a**) Bet v 1a, (**b**) Art v 1, (**c**) Phl p 5b. Cultivars: A—Marynka, B—Lubelski, C—Magnum. Plant parts: 1—stalk, 2—leaves, 3—cone. MWM—molecular weight marker. Asterisks denote detected fractions.

Western Blotting was employed to identify immunoreactive protein fractions in hop cones, leaves, and stalks using antibodies specific to Bet v 1, Art v 1, and Phl p 5 (Figure 4).

Among the samples, the Bet v 1-specific IgG recognized a 19 kDa protein in the cones of the Marynka cultivar, as well as in the cones and stalks of the Magnum cultivar. Similarly, the Phl p 5-specific IgG detected a 19 kDa protein in the cones of all three cultivars. In contrast, the Art v 1-specific IgG recognized multiple protein fractions across all samples, ranging from 15 kDa in the cones to 68 kDa in the stalks of Magnum cultivar.

**Figure 5:**
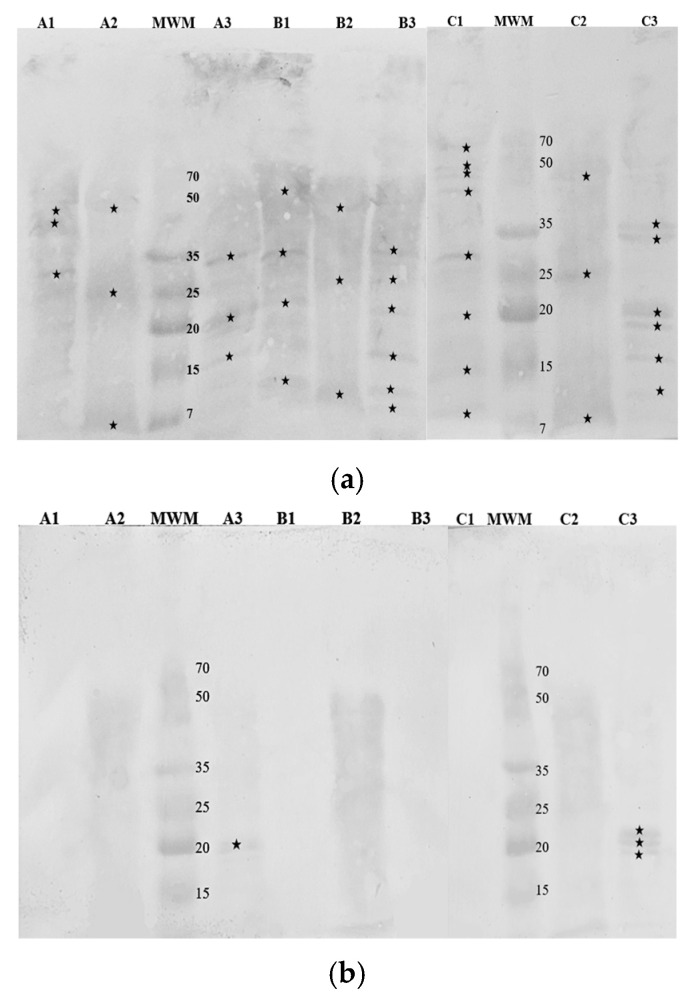

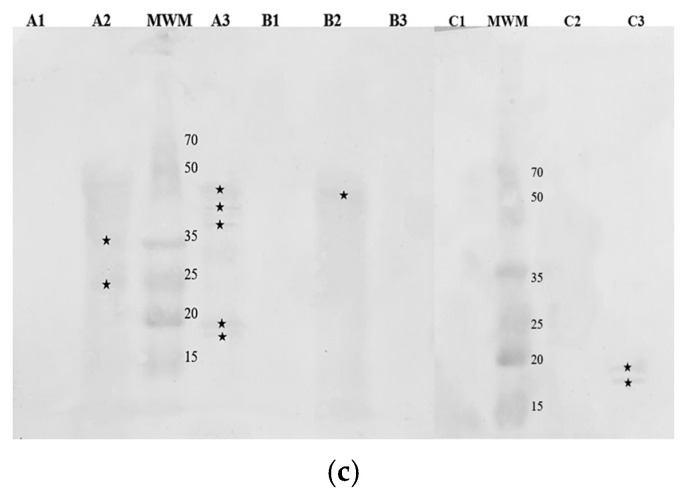
Membranes obtained after immunoblotting of hop samples with sera from allergenic patients: (**a**) serum 1, (**b**) serum 2, (**c**) serum 3. Cultivars: A—Marynka, B—Lubelski, C—Magnum. Plant parts: 1—stalk, 2—leaves, 3—cone. MWM—molecular weight marker. Asterisks denote detected fractions.

Immunoreactivity was further confirmed using patient sera, as demonstrated in Figure 5.

Serum 1, from a patient with high sensitivity to birch pollen (class 6), reacted with several proteins across all hop samples, particularly in cones and stalks, where protein fractions between 7 and 65 kDa were detected. Notable fractions at 35 kDa, 19 kDa, and 15 kDa were consistently identified in the cones of all cultivars. The Magnum cultivar showed additional immunoreactive bands at 33 kDa, 18 kDa, and 10 kDa while the Lubelski cultivar had proteins at 25 kDa, 10 kDa, and 7 kDa. According to the WHO/IUIS Allergen Nomenclature Subcommittee [17], nine birch allergens have been identified with molecular weights of 7–8 kDa, 14 kDa, 15 kDa, 17 kDa, 18 kDa, 24 kDa, 27 kDa, and 35 kDa. The Western Blot patterns of immunoreactive hop proteins with serum 1 IgE revealed similar molecular weights.

Serum 2, from a patient with high sensitivity to mugwort (class 4), reacted primarily with cone proteins from the Magnum and Marynka cultivars (Figure 5b), with fractions around 20 kDa, similar to the mugwort allergen Art v 2. Additional proteins of 18 kDa and 19 kDa were detected in the Magnum cultivar, showing molecular weight similarities to allergenic proteins from apples, strawberries, and peanuts, to which the patient was also allergic. Hop leaves exhibited weak reactivity with serum 2, though the specific molecular weights of the reactive fractions were not determined. No reactivity was observed with stalk proteins.

Serum 3, from a patient with moderate sensitivity to grass pollen (class 2), also showed immunoreactivity with hop cone proteins (Figure 5c), particularly in the Marynka cultivar, with molecular weights at 18 kDa, 19 kDa, 40 kDa, 45 kDa, and 48 kDa. In the Magnum cultivar, proteins at 18 kDa and 19 kDa were detected. Hop leaves from the Marynka cultivar showed reactivity at 25 kDa and 35 kDa, while leaves from the Lubelski cultivar displayed proteins at 48 kDa. According to the WHO/IUIS Subcommittee, 10 airborne mugwort allergens have been identified, including Phl p 1 (27 kDa), Phl p 2 (10–12 kDa), Phl p 3 (10.9 kDa), Phl p 4 (55 kDa), Phl p 5 (32 kDa), Phl p 6 (11 kDa), Phl p 7 (6 kDa), Phl p 11 (20 kDa), Phl p 12 (14 kDa), and Phl p 13 (55 kDa).

## 4. Discussion

In this preliminary study, we investigated the immunoreactivity of proteins extracted from cones, leaves, and stalks of three hop cultivars (Marynka, Lubelski Magnum), with the goal of assessing their potential cross-reactivity with common pollen allergens such as birch (Bet v 1), mugwort (Art v 1), and timothy grass (Phl p 5). Birch (*Betula pendula* Roth), mugwort (*Artemisia vulgaris* L), and timothy grass (*Phleum pratense* L.) were selected as representative allergens from frequently sensitizing tree, weed, and grass pollens, each belonging to different protein families [40,41,42]. Wu et al. [40] reported that 88.8% of patients with allergic rhinitis or asthma tested positive for mugwort-specific IgE, 30% for timothy, and 32.5% for birch-specific IgE. It is important to note that Bet v 1, Art v 1, and Phl p 5 are panallergens that often cause cross-reactive pollen–pollen and pollen–food reactions [30,43].

The immunoreactivity of hop proteins observed in our study aligns with previous findings from García et al. [10], who reported cross-reactivity between grass pollen and hop allergens in brewery workers. Similarly, Li et al. [44] observed cross-reactions between tree pollen allergens, ragweed, and *Humulus japonicus*. Our study extends these findings by identifying specific protein fractions in hop cones and stalks that may share epitopes with major pollen allergens. The strong reactivity observed for the cone extracts of all cultivars suggests that hop cones could be particularly potent sources of cross-reactive allergens. This hypothesis was supported by the Spiewak and Dudkiewicz study [12], who noted that extracts from hop cones elicited stronger allergic responses in skin prick tests compared to leaf extracts.

The immunoreactivity of hop proteins with antibodies specific to Bet v 1, Art v 1, and Phl p 5, as well as sera from allergic patients, suggests that hop proteins may act as panallergens, capable of cross-reacting with proteins even from unrelated plant species. This observation is in line with studies on panallergens, such as those by Wu et al. [40], which highlight the role of PR-10 proteins (like Bet v1) in cross-reactive allergic reactions. Approximately 70% of individuals allergic to birch pollen also experience oral allergy syndrome triggered by homologous proteins in fruits and vegetables, such as those found in the Rosaceae family (e.g., apples, cherries) [25]. Our identification of a 19 kDa protein in hop cones and stalks, which was recognized by Bet v 1-specific antibodies, suggests that similar cross-reactions could occur in individuals with birch pollen allergies who are exposed to hop proteins.

The strong reactivity of hop proteins with Art v 1-specific commercial IgG as well as IgE in mugwort allergic patient sera further supports the hypothesis that hops contain proteins capable of cross-reacting with weed pollen allergens. Similar findings have been reported by Li et al. [44], who observed cross-reactivity between *Humulus japonicus* and ragweed (*Ambrosia* spp.), a major allergen in North America. Our results, which demonstrate that hop proteins react with serum from mugwort-allergic patients, are consistent with these observations. The Art v 1 protein, with its unique polyproline-rich tail, is known to share epitopes with other plant proteins such as Amb a 1 from ragweed and Par h 1 from fever [45], Api g 7 from celery [46] and others. The detection of multiple immunoreactive proteins in hop extracts, particularly those recognized by Art v 1-specific antibodies, suggests that hops could be an important source of cross-reactive allergens for individuals sensitized to weed pollen. However, it is important to note that the Art v 1 antibodies used in this study were produced through immunization with recombinant proteins, which may exhibit a higher tendency to cross-react compared to antibodies generated from purified proteins. This is primarily because recombinant proteins can contain additional epitopes that may not be present in the native purified protein. Consequently, the use of such antibodies increases the likelihood of cross-reactivity and non-specific signals, as they may recognize multiple epitopes.

The detection of proteins in the 19–20 kDa range across all hop cultivars, recognized by Phl p 5-specific antibodies, is particularly interesting in the context of grass pollen allergies. Previous studies have shown that Phl p 5 is a major timothy grass allergen with strong cross-reactivity potential due to its conserved structure, which includes two domains connected by a flexible linker region of cross-reactive allergens also for individuals sensitized to weed pollens [47,48]. The recognition of similar-sized proteins in hops suggests that hop proteins may share structural features with Phl p 5, leading to cross-reactions in individuals sensitized to grass pollen.

Our results indicate that hop proteins, particularly those found in cones and stalks, exhibit strong immunoreactivity and may serve as panallergens capable of triggering cross-reactive allergic responses in individuals sensitized to common pollen allergens. This has important implications for the use of hop-based products, such as dietary supplements, cosmetics, and beverages. Previous studies have shown that patients with pollen allergies may experience allergic reactions to plant-based products containing cross-reactive proteins. For example, Bet v 1-homologous proteins in fruits and vegetables have been identified as major contributors to pollen food syndrome [49].

While denaturation during Western Blotting reduced the immunoreactivity of hop proteins, indicating that processing may diminish allergenic potential, this does not fully eliminate the risk. Native (non-denatured) hop proteins, which retain strong immunoreactivity, could pose a risk to allergic individuals using hop-containing products. Since dietary supplements typically contain native hop proteins, the risk of allergic reactions for pollen-sensitive individuals remains a concern. Our results suggest that hop proteins may function as panallergens, similar to pollen proteins, potentially causing unexpected allergic reactions in individuals with pollen allergies who consume hop-containing supplements (e.g., to improve sleep) or use cosmetics. Since these products are marked as “natural remedies”, consumers may not even expect an allergenic reaction.

Although this preliminary study provides valuable insights into the immunoreactive properties of hop proteins, further research is needed to fully understand the mechanisms of cross-reactivity and to identify the specific proteins responsible for these reactions. Discrepancies between the Slot Blot and Western Blot analyses suggest that additional factors, such as protein conformation and post-translational modifications, may influence allergen recognition. Future studies should focus on the molecular characterization of hop proteins and explore strategies for reducing the allergenic potential of hop-based products.

## 5. Conclusions

The preliminary study demonstrates that hop proteins are recognized by antibodies specific to major pollen allergens (Bet v1, Art v2, and Phl p 5) and by IgE from individuals sensitized to birch, mugwort, and timothy grass pollen. Although denaturation reduces the immunoreactivity of hop proteins, it does not eliminate their allergenic potential. These findings suggest that hop proteins, particularly in their native form, may act as cross-reactive allergens, posing a risk to individuals with pollen allergies.

Given the increasing use of hops in dietary supplements and other products, there is a need for heightened awareness of their potential allergenicity. Further research is crucial to better understand the mechanisms of cross-reactivity and to identify specific hop proteins that may contribute to allergic reactions in pollen-sensitized individuals. The development of strategies to mitigate these risks, especially in products containing native hop proteins, should guide future studies.

## Figures and Tables

**Table 1:** Allergenic characteristics of patients’ sera.

Serum	Allergens	Class of Allergy
1	Birch pollen	6
	Hazel pollen	2
	Grass mix pollen	1
2	Mugwort pollen	4
	Grape	3
	Apple	3
	Peanuts	2
	Walnut	2
	Strawberries	2
3	Grass pollen mix	2
	Bovine serum albumin	2
	Pork	1

**Table 2:** Average protein concentrations of the extracts from stalks, leaves, and cones of three hop cultivars [μg/mL].

Plant Part	Hop Cultivar		
	Marynka	Lubelski	Magnum
Stalk	92 ± 4.1 ^b^	81 ± 5.6 ^c^	58 ± 4.2 ^e^
Leaves	40 ± 2.9 ^f^	53 ± 2.3 ^e^	72 ± 3.5 ^d^
Cones	87 ± 3.1 ^c^	119 ± 4.4 ^a^	96 ± 3.7 ^b^

Note: a, b, c, d, e, f values sharing the same letter indicate no significant statistical differences between hop extracts (*p* = 0.05).

## Data Availability

The original contributions presented in the study are included in the article, further inquiries can be directed to the corresponding author.
